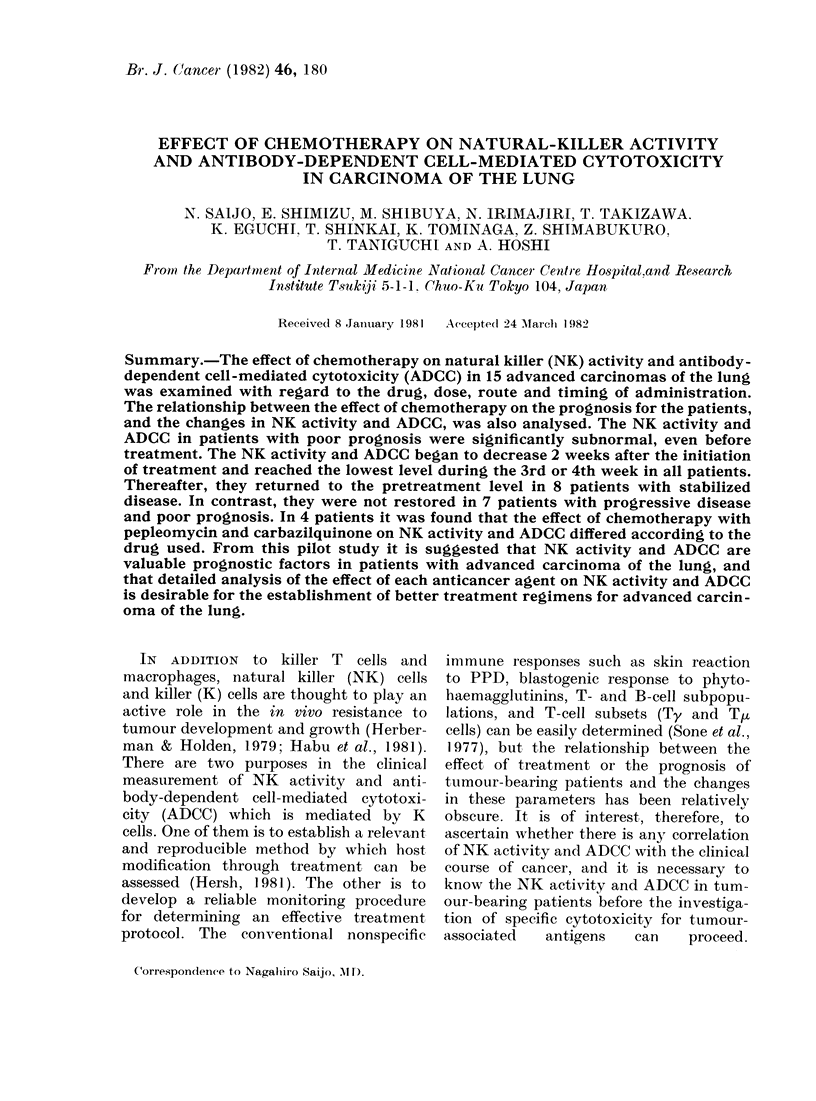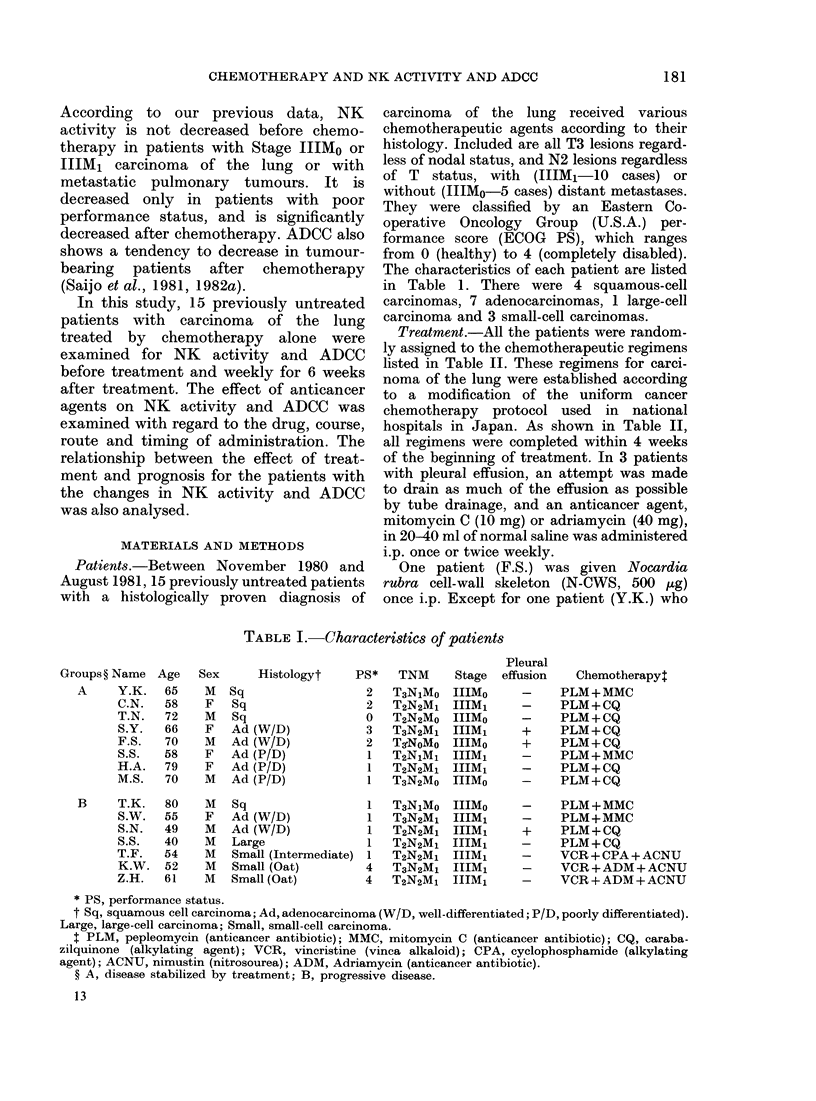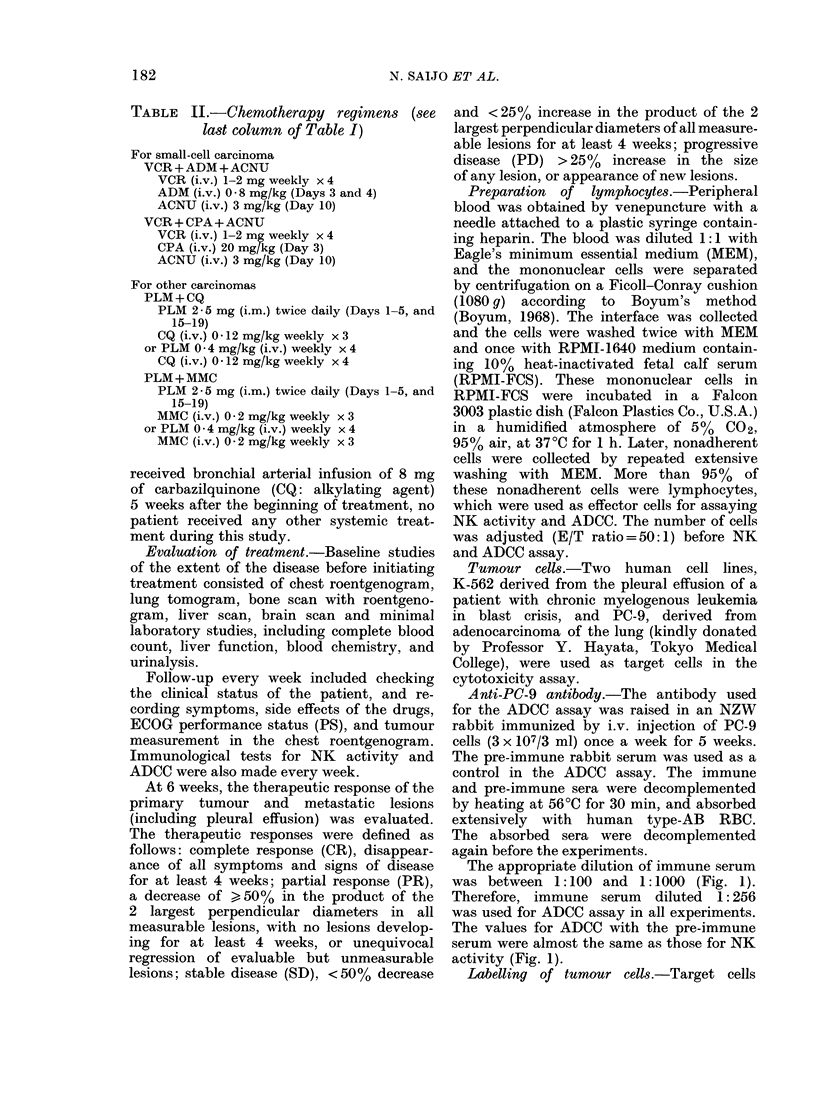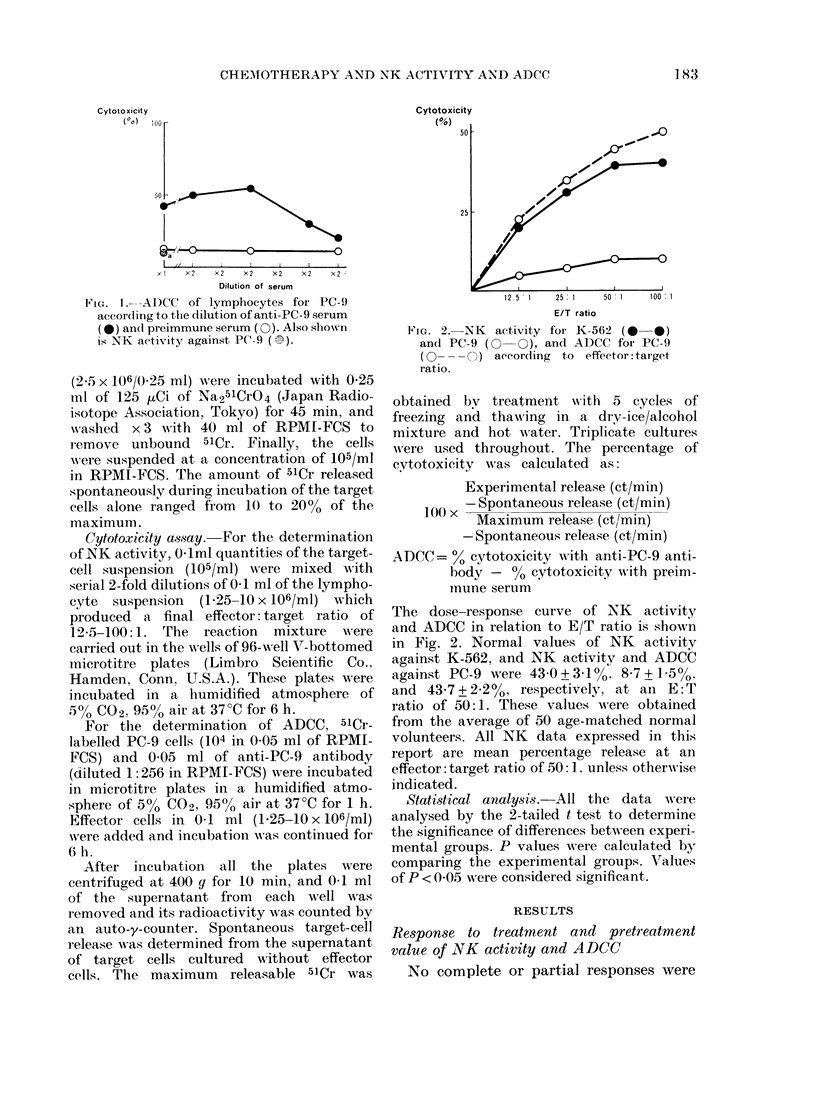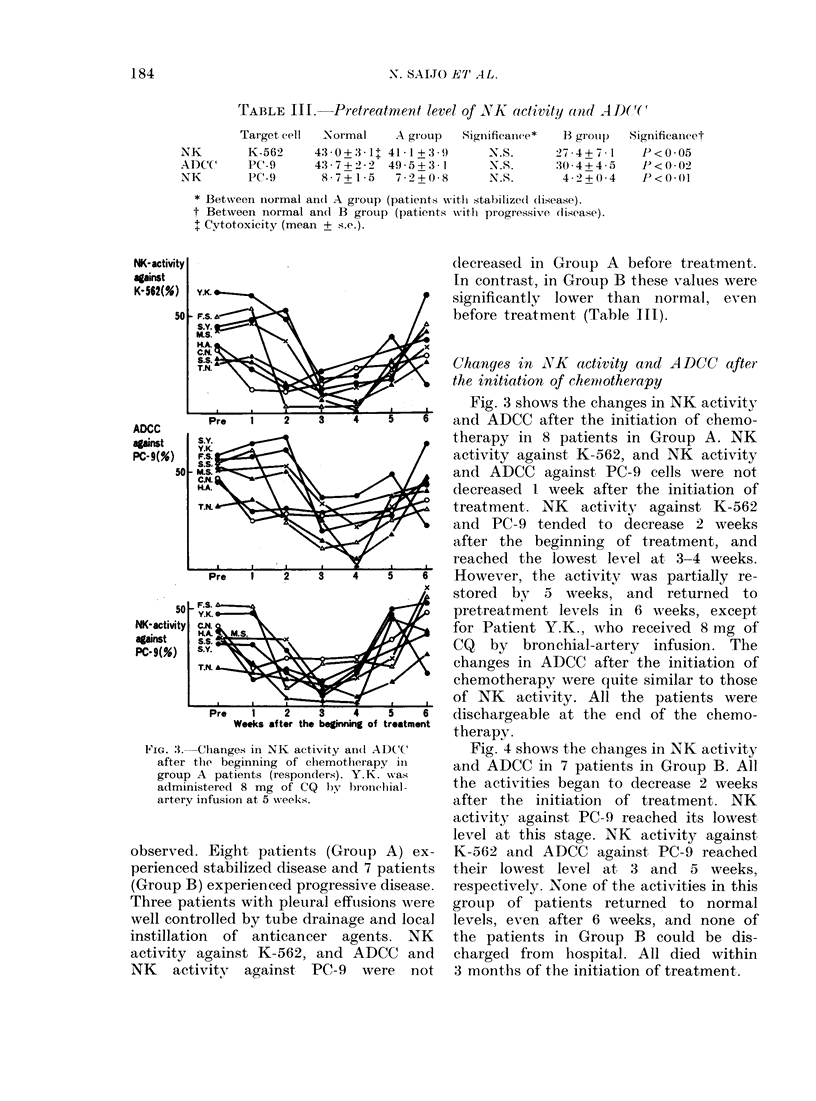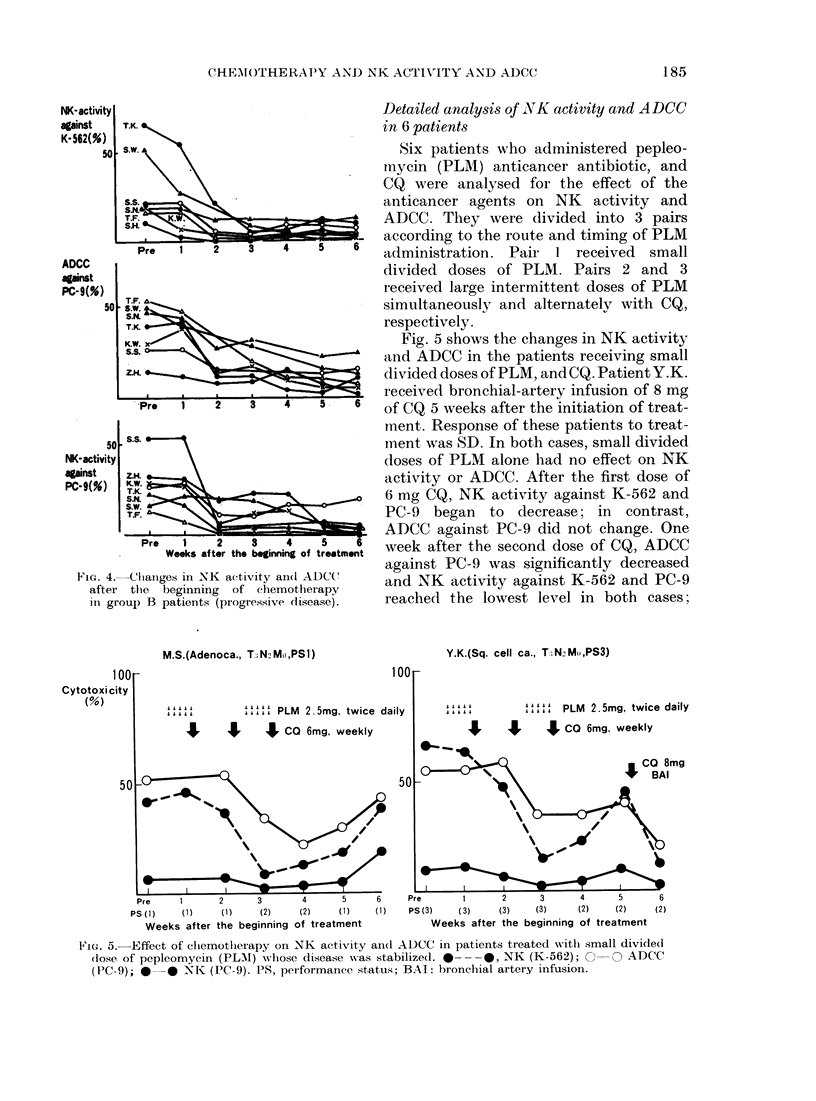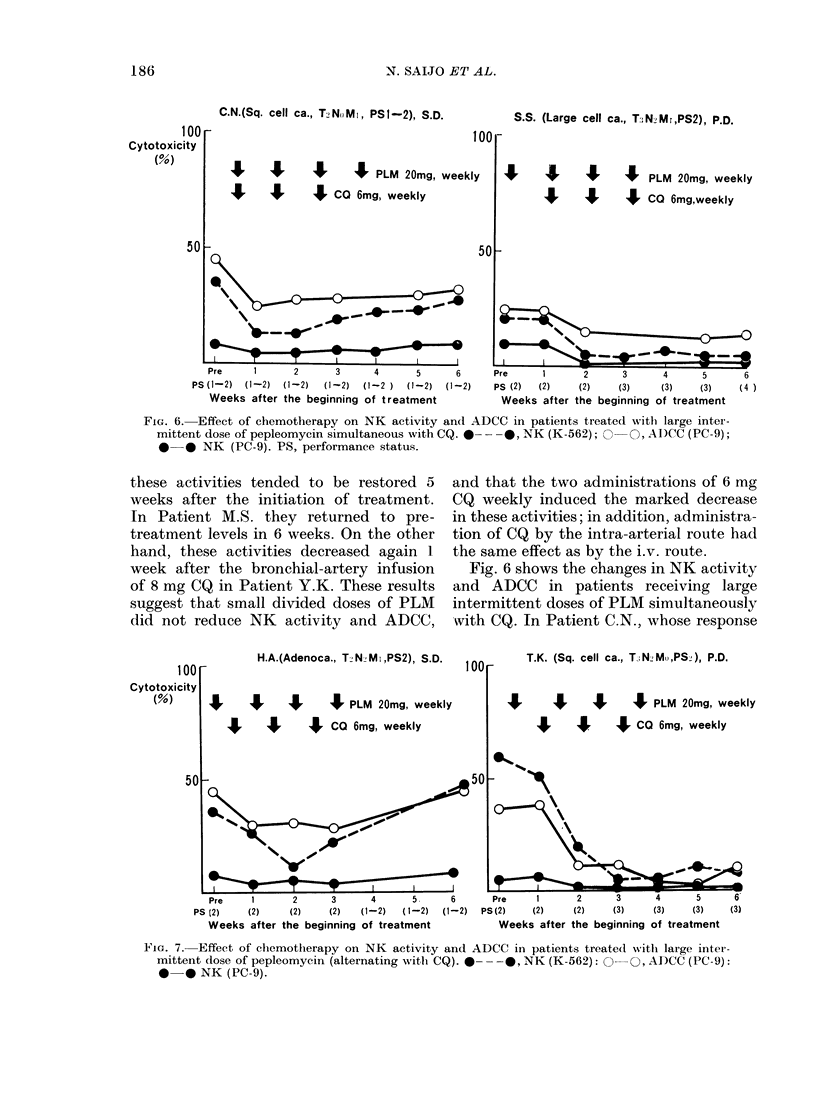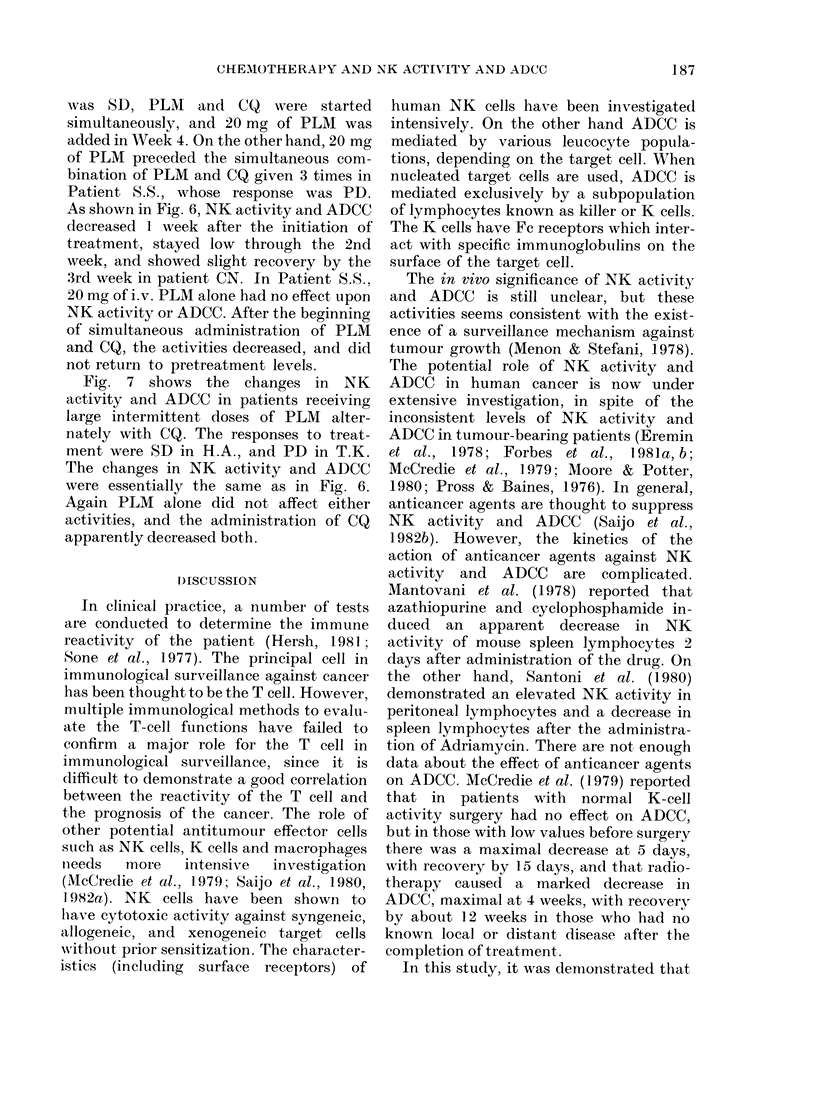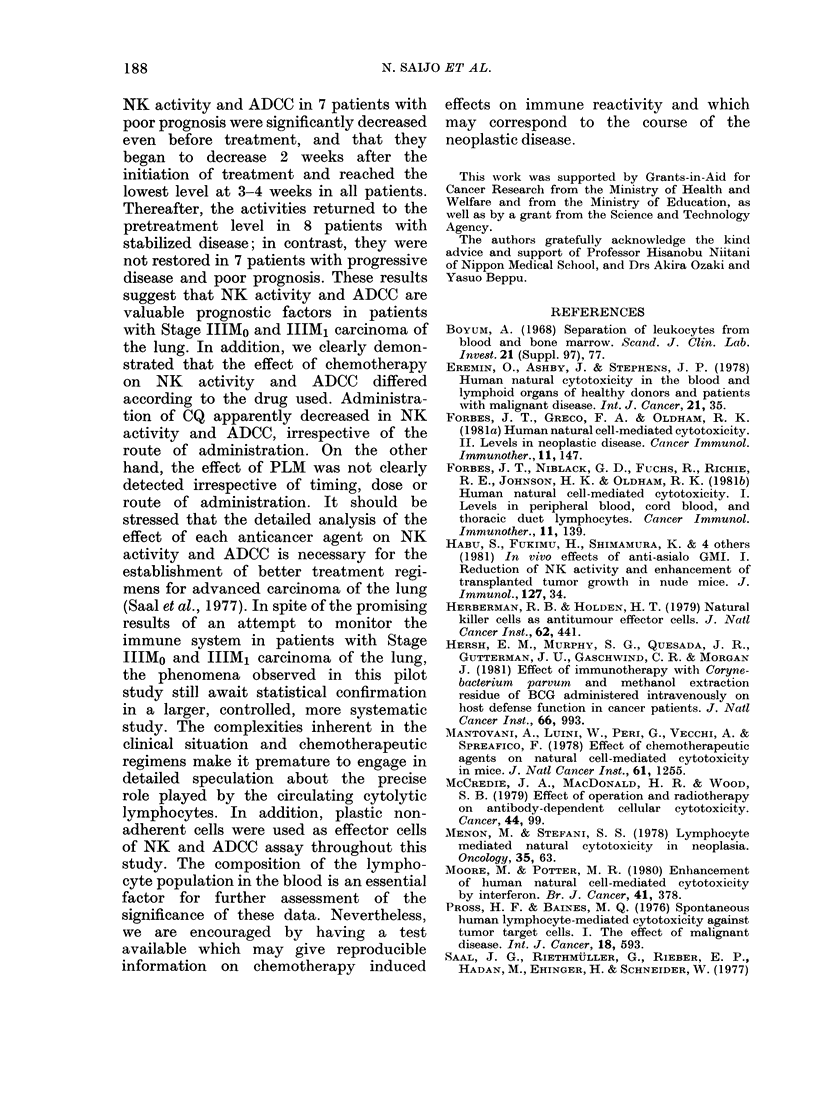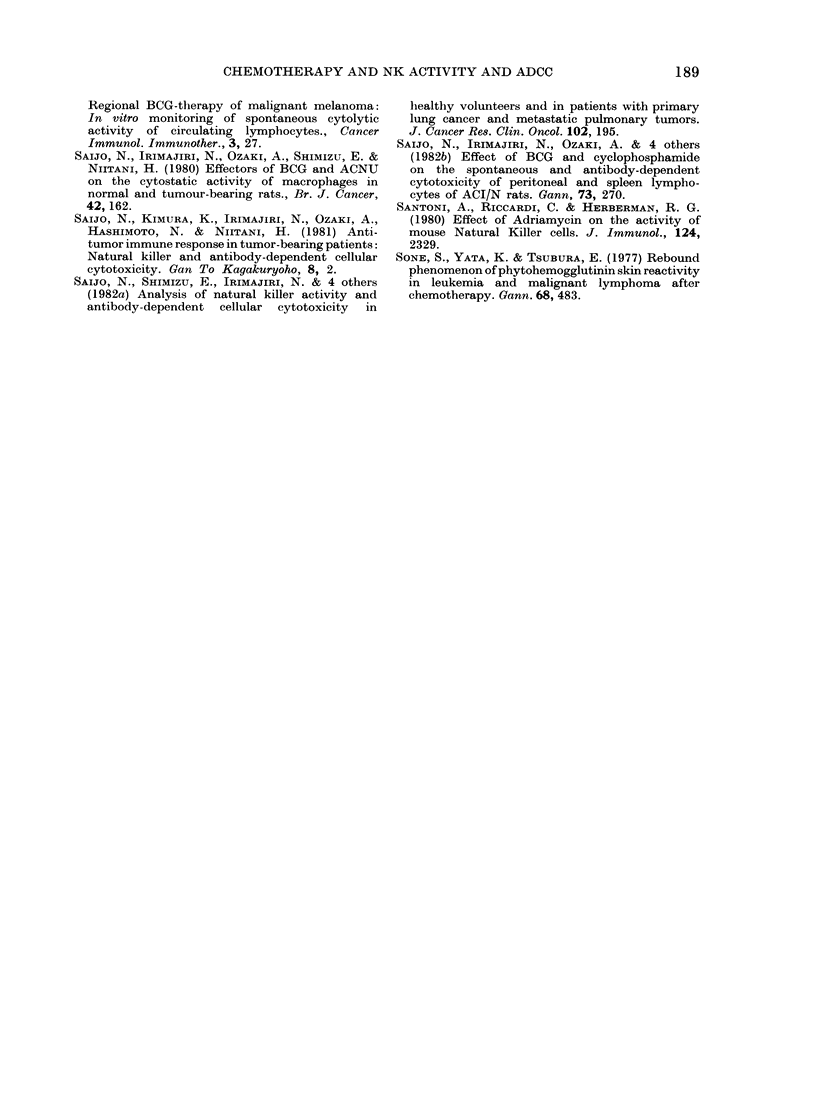# Effect of chemotherapy on natural-killer activity and antibody-dependent cell-mediated cytotoxicity in carcinoma of the lung.

**DOI:** 10.1038/bjc.1982.182

**Published:** 1982-08

**Authors:** N. Saijo, E. Shimizu, M. Shibuya, N. Irimajiri, T. Takizawa, K. Eguchi, T. Shinkai, K. Tominaga, Z. Shimabukuro, T. Taniguchi, A. Hoshi

## Abstract

The effect of chemotherapy on natural killer (NK) activity and antibody-dependent cell-mediated cytotoxicity (ADCC) in 15 advanced carcinomas of the lung was examined with regard to the drug, dose, route and timing of administration. The relationship between the effect of chemotherapy on the prognosis for the patients, and the changes in NK activity and ADCC, was also analysed. The NK activity and ADCC in patients with poor prognosis were significantly subnormal, even before treatment. The NK activity and ADCC began to decrease 2 weeks after the initiation of treatment and reached the lowest level during the 3rd or 4th week in all patients. Thereafter, they returned to the pretreatment level in 8 patients with stabilized disease. In contrast, they were not restored in 7 patients with progressive disease and poor prognosis. In 4 patients it was found that the effect of chemotherapy with pepleomycin and carbazilquinone on NK activity and ADCC differed according to the drug used. From this pilot study it is suggested that NK activity and ADCC are valuable prognostic factors in patients with advanced carcinoma of the lung, and that detailed analysis of the effect of each anticancer agent on NK activity and ADCC is desirable for the establishment of better treatment regimens for advanced carcinoma of the lung.


*Br. J. Cancer* (1982) **46**, 180

### EFFECT OF CHEMOTHERAPY ON NATURAL-KILLER ACTIVITY AND ANTIBODY-DEPENDENT CELL-MEDIATED CYTOTOXICITY IN CARCINOMA OF THE LUNG

N. SAIJO, E. SHIMIZU, M. SHIBUYA, N. IRIMAJIRI, T. TAKIZAWA, K. EGUCHI, T. SHINKAI, K. TOMINAGA, Z. SHIMABUKURO, T. TANIGUCHI AND A. HOSHI

*From the Department of Internal Medicine National Cancer Centre Hospital, and Research Institute Tsukiji* 5-1-1, *Chuo-Ku Tokyo* 104, *Japan*

Received 8 January 1981 Accepted 24 March 1982

**Summary.—The effect of chemotherapy on natural killer (NK) activity and antibody-dependent cell-mediated cytotoxicity (ADCC) in 15 advanced carcinomas of the lung was examined with regard to the drug, dose, route and timing of administration. The relationship between the effect of chemotherapy on the prognosis for the patients, and the changes in NK activity and ADCC, was also analysed. The NK activity and ADCC in patients with poor prognosis were significantly subnormal, even before treatment. The NK activity and ADCC began to decrease 2 weeks after the initiation of treatment and reached the lowest level during the 3rd or 4th week in all patients. Thereafter, they returned to the pretreatment level in 8 patients with stabilized disease. In contrast, they were not restored in 7 patients with progressive disease and poor prognosis. In 4 patients it was found that the effect of chemotherapy with pepleomycin and carbazilquinone on NK activity and ADCC differed according to the drug used. From this pilot study it is suggested that NK activity and ADCC are valuable prognostic factors in patients with advanced carcinoma of the lung, and that detailed analysis of the effect of each anticancer agent on NK activity and ADCC is desirable for the establishment of better treatment regimens for advanced carcinoma of the lung.**

IN ADDITION to killer T cells and macrophages, natural killer (NK) cells and killer (K) cells are thought to play an active role in the *in vivo* resistance to tumour development and growth (Herberman & Holden, 1979; Habu *et al.*, 1981). There are two purposes in the clinical measurement of NK activity and antibody-dependent cell-mediated cytotoxicity (ADCC) which is mediated by K cells. One of them is to establish a relevant and reproducible method by which host modification through treatment can be assessed (Hersh, 1981). The other is to develop a reliable monitoring procedure for determining an effective treatment protocol. The conventional nonspecific immune responses such as skin reaction to PPD, blastogenic response to phytohaemagglutinins, T- and B-cell subpopulations, and T-cell subsets ($T\gamma$ and $T\mu$ cells) can be easily determined (Sone *et al.*, 1977), but the relationship between the effect of treatment or the prognosis of tumour-bearing patients and the changes in these parameters has been relatively obscure. It is of interest, therefore, to ascertain whether there is any correlation of NK activity and ADCC with the clinical course of cancer, and it is necessary to know the NK activity and ADCC in tumour-bearing patients before the investigation of specific cytotoxicity for tumour-associated antigens can proceed.

Correspondence to Nagahiro Saijo, MD.

According to our previous data, NK activity is not decreased before chemotherapy in patients with Stage IIIM$_0$ or IIIM$_1$ carcinoma of the lung or with metastatic pulmonary tumours. It is decreased only in patients with poor performance status, and is significantly decreased after chemotherapy. ADCC also shows a tendency to decrease in tumour-bearing patients after chemotherapy (Saijo *et al.*, 1981, 1982*a*).

In this study, 15 previously untreated patients with carcinoma of the lung treated by chemotherapy alone were examined for NK activity and ADCC before treatment and weekly for 6 weeks after treatment. The effect of anticancer agents on NK activity and ADCC was examined with regard to the drug, course, route and timing of administration. The relationship between the effect of treatment and prognosis for the patients with the changes in NK activity and ADCC was also analysed.

### MATERIALS AND METHODS

*Patients*.—Between November 1980 and August 1981, 15 previously untreated patients with a histologically proven diagnosis of carcinoma of the lung received various chemotherapeutic agents according to their histology. Included are all T3 lesions regardless of nodal status, and N2 lesions regardless of T status, with (IIIM$_1$—10 cases) or without (IIIM$_0$—5 cases) distant metastases. They were classified by an Eastern Co-operative Oncology Group (U.S.A.) performance score (ECOG PS), which ranges from 0 (healthy) to 4 (completely disabled). The characteristics of each patient are listed in Table 1. There were 4 squamous-cell carcinomas, 7 adenocarcinomas, 1 large-cell carcinoma and 3 small-cell carcinomas.

*Treatment*.—All the patients were randomly assigned to the chemotherapeutic regimens listed in Table II. These regimens for carcinoma of the lung were established according to a modification of the uniform cancer chemotherapy protocol used in national hospitals in Japan. As shown in Table II, all regimens were completed within 4 weeks of the beginning of treatment. In 3 patients with pleural effusion, an attempt was made to drain as much of the effusion as possible by tube drainage, and an anticancer agent, mitomycin C (10 mg) or adriamycin (40 mg), in 20–40 ml of normal saline was administered i.p. once or twice weekly.

One patient (F.S.) was given *Nocardia rubra* cell-wall skeleton (N-CWS, 500 $\mu$g) once i.p. Except for one patient (Y.K.) who

TABLE I.—*Characteristics of patients*

| Groups§ | Name | Age | Sex | Histology† | PS* | TNM | Stage | Pleural effusion | Chemotherapy‡ |
|---|---|---|---|---|---|---|---|---|---|
| A | Y.K. | 65 | M | Sq | 2 | $T_3N_1M_0$ | IIIM$_0$ | − | PLM+MMC |
| | C.N. | 58 | F | Sq | 2 | $T_2N_2M_1$ | IIIM$_1$ | − | PLM+CQ |
| | T.N. | 72 | M | Sq | 0 | $T_2N_2M_0$ | IIIM$_0$ | − | PLM+CQ |
| | S.Y. | 66 | F | Ad (W/D) | 3 | $T_3N_2M_1$ | IIIM$_1$ | + | PLM+CQ |
| | F.S. | 70 | M | Ad (W/D) | 2 | $T_3N_0M_0$ | IIIM$_0$ | + | PLM+CQ |
| | S.S. | 58 | F | Ad (P/D) | 1 | $T_2N_1M_1$ | IIIM$_1$ | − | PLM+MMC |
| | H.A. | 79 | F | Ad (P/D) | 1 | $T_2N_2M_1$ | IIIM$_1$ | − | PLM+CQ |
| | M.S. | 70 | M | Ad (P/D) | 1 | $T_3N_2M_0$ | IIIM$_0$ | − | PLM+CQ |
| B | T.K. | 80 | M | Sq | 1 | $T_3N_1M_0$ | IIIM$_0$ | − | PLM+MMC |
| | S.W. | 55 | F | Ad (W/D) | 1 | $T_3N_2M_1$ | IIIM$_1$ | − | PLM+MMC |
| | S.N. | 49 | M | Ad (W/D) | 1 | $T_2N_2M_1$ | IIIM$_1$ | + | PLM+CQ |
| | S.S. | 40 | M | Large | 1 | $T_2N_2M_1$ | IIIM$_1$ | − | PLM+CQ |
| | T.F. | 54 | M | Small (Intermediate) | 1 | $T_2N_2M_1$ | IIIM$_1$ | − | VCR+CPA+ACNU |
| | K.W. | 52 | M | Small (Oat) | 4 | $T_3N_2M_1$ | IIIM$_1$ | − | VCR+ADM+ACNU |
| | Z.H. | 61 | M | Small (Oat) | 4 | $T_2N_2M_1$ | IIIM$_1$ | − | VCR+ADM+ACNU |

* PS, performance status.

† Sq, squamous cell carcinoma; Ad, adenocarcinoma (W/D, well-differentiated; P/D, poorly differentiated). Large, large-cell carcinoma; Small, small-cell carcinoma.

‡ PLM, pepleomycin (anticancer antibiotic); MMC, mitomycin C (anticancer antibiotic); CQ, carabazilquinone (alkylating agent); VCR, vincristine (vinca alkaloid); CPA, cyclophosphamide (alkylating agent); ACNU, nimustin (nitrosourea); ADM, Adriamycin (anticancer antibiotic).

§ A, disease stabilized by treatment; B, progressive disease.

TABLE II.—*Chemotherapy regimens (see last column of Table I)*

For small-cell carcinoma
- VCR+ADM+ACNU
  - VCR (i.v.) 1–2 mg weekly ×4
  - ADM (i.v.) 0·8 mg/kg (Days 3 and 4)
  - ACNU (i.v.) 3 mg/kg (Day 10)
- VCR+CPA+ACNU
  - VCR (i.v.) 1–2 mg weekly ×4
  - CPA (i.v.) 20 mg/kg (Day 3)
  - ACNU (i.v.) 3 mg/kg (Day 10)

For other carcinomas
- PLM+CQ
  - PLM 2·5 mg (i.m.) twice daily (Days 1–5, and 15–19)
  - CQ (i.v.) 0·12 mg/kg weekly ×3
  - or PLM 0·4 mg/kg (i.v.) weekly ×4
  - CQ (i.v.) 0·12 mg/kg weekly ×4
- PLM+MMC
  - PLM 2·5 mg (i.m.) twice daily (Days 1–5, and 15–19)
  - MMC (i.v.) 0·2 mg/kg weekly ×3
  - or PLM 0·4 mg/kg (i.v.) weekly ×4
  - MMC (i.v.) 0·2 mg/kg weekly ×3

received bronchial arterial infusion of 8 mg of carbazilquinone (CQ: alkylating agent) 5 weeks after the beginning of treatment, no patient received any other systemic treatment during this study.

*Evaluation of treatment.*—Baseline studies of the extent of the disease before initiating treatment consisted of chest roentgenogram, lung tomogram, bone scan with roentgenogram, liver scan, brain scan and minimal laboratory studies, including complete blood count, liver function, blood chemistry, and urinalysis.

Follow-up every week included checking the clinical status of the patient, and recording symptoms, side effects of the drugs, ECOG performance status (PS), and tumour measurement in the chest roentgenogram. Immunological tests for NK activity and ADCC were also made every week.

At 6 weeks, the therapeutic response of the primary tumour and metastatic lesions (including pleural effusion) was evaluated. The therapeutic responses were defined as follows: complete response (CR), disappearance of all symptoms and signs of disease for at least 4 weeks; partial response (PR), a decrease of $\geqslant 50\%$ in the product of the 2 largest perpendicular diameters in all measurable lesions, with no lesions developing for at least 4 weeks, or unequivocal regression of evaluable but unmeasurable lesions; stable disease (SD), $<50\%$ decrease and $<25\%$ increase in the product of the 2 largest perpendicular diameters of all measureable lesions for at least 4 weeks; progressive disease (PD) $>25\%$ increase in the size of any lesion, or appearance of new lesions.

*Preparation of lymphocytes.*—Peripheral blood was obtained by venepuncture with a needle attached to a plastic syringe containing heparin. The blood was diluted 1:1 with Eagle's minimum essential medium (MEM), and the mononuclear cells were separated by centrifugation on a Ficoll–Conray cushion (1080 *g*) according to Boyum's method (Boyum, 1968). The interface was collected and the cells were washed twice with MEM and once with RPMI-1640 medium containing 10% heat-inactivated fetal calf serum (RPMI-FCS). These mononuclear cells in RPMI-FCS were incubated in a Falcon 3003 plastic dish (Falcon Plastics Co., U.S.A.) in a humidified atmosphere of 5% $CO_2$, 95% air, at 37°C for 1 h. Later, nonadherent cells were collected by repeated extensive washing with MEM. More than 95% of these nonadherent cells were lymphocytes, which were used as effector cells for assaying NK activity and ADCC. The number of cells was adjusted (E/T ratio=50:1) before NK and ADCC assay.

*Tumour cells.*—Two human cell lines, K-562 derived from the pleural effusion of a patient with chronic myelogenous leukemia in blast crisis, and PC-9, derived from adenocarcinoma of the lung (kindly donated by Professor Y. Hayata, Tokyo Medical College), were used as target cells in the cytotoxicity assay.

*Anti-PC-9 antibody.*—The antibody used for the ADCC assay was raised in an NZW rabbit immunized by i.v. injection of PC-9 cells ($3\times10^7$/3 ml) once a week for 5 weeks. The pre-immune rabbit serum was used as a control in the ADCC assay. The immune and pre-immune sera were decomplemented by heating at 56°C for 30 min, and absorbed extensively with human type-AB RBC. The absorbed sera were decomplemented again before the experiments.

The appropriate dilution of immune serum was between 1:100 and 1:1000 (Fig. 1). Therefore, immune serum diluted 1:256 was used for ADCC assay in all experiments. The values for ADCC with the pre-immune serum were almost the same as those for NK activity (Fig. 1).

*Labelling of tumour cells.*—Target cells

FIG. 1.—ADCC of lymphocytes for PC-9 according to the dilution of anti-PC-9 serum (●) and preimmune serum (○). Also shown is NK activity against PC-9 (◍).

FIG. 2.—NK activity for K-562 (●—●) and PC-9 (○—○), and ADCC for PC-9 (○- - -○) according to effector:target ratio.

($2{\cdot}5\times10^6/0{\cdot}25$ ml) were incubated with 0·25 ml of 125 $\mu$Ci of $Na_2{}^{51}CrO_4$ (Japan Radioisotope Association, Tokyo) for 45 min, and washed ×3 with 40 ml of RPMI-FCS to remove unbound $^{51}$Cr. Finally, the cells were suspended at a concentration of $10^5$/ml in RPMI-FCS. The amount of $^{51}$Cr released spontaneously during incubation of the target cells alone ranged from 10 to 20% of the maximum.

*Cytotoxicity assay.*—For the determination of NK activity, 0·1ml quantities of the target-cell suspension ($10^5$/ml) were mixed with serial 2-fold dilutions of 0·1 ml of the lymphocyte suspension ($1{\cdot}25$–$10\times10^6$/ml) which produced a final effector: target ratio of 12·5–100:1. The reaction mixture were carried out in the wells of 96-well V-bottomed microtitre plates (Limbro Scientific Co., Hamden, Conn, U.S.A.). These plates were incubated in a humidified atmosphere of 5% $CO_2$, 95% air at 37°C for 6 h.

For the determination of ADCC, $^{51}$Cr-labelled PC-9 cells ($10^4$ in 0·05 ml of RPMI-FCS) and 0·05 ml of anti-PC-9 antibody (diluted 1:256 in RPMI-FCS) were incubated in microtitre plates in a humidified atmosphere of 5% $CO_2$, 95% air at 37°C for 1 h. Effector cells in 0·1 ml ($1{\cdot}25$–$10\times10^6$/ml) were added and incubation was continued for 6 h.

After incubation all the plates were centrifuged at 400 $g$ for 10 min, and 0·1 ml of the supernatant from each well was removed and its radioactivity was counted by an auto-$\gamma$-counter. Spontaneous target-cell release was determined from the supernatant of target cells cultured without effector cells. The maximum releasable $^{51}$Cr was obtained by treatment with 5 cycles of freezing and thawing in a dry-ice/alcohol mixture and hot water. Triplicate cultures were used throughout. The percentage of cytotoxicity was calculated as:

$$100\times\frac{\text{Experimental release (ct/min)}-\text{Spontaneous release (ct/min)}}{\text{Maximum release (ct/min)}-\text{Spontaneous release (ct/min)}}$$

ADCC = % cytotoxicity with anti-PC-9 antibody − % cytotoxicity with preimmune serum

The dose–response curve of NK activity and ADCC in relation to E/T ratio is shown in Fig. 2. Normal values of NK activity against K-562, and NK activity and ADCC against PC-9 were $43{\cdot}0\pm3{\cdot}1$%, $8{\cdot}7\pm1{\cdot}5$%, and $43{\cdot}7\pm2{\cdot}2$%, respectively, at an E:T ratio of 50:1. These values were obtained from the average of 50 age-matched normal volunteers. All NK data expressed in this report are mean percentage release at an effector:target ratio of 50:1, unless otherwise indicated.

*Statistical analysis.*—All the data were analysed by the 2-tailed $t$ test to determine the significance of differences between experimental groups. $P$ values were calculated by comparing the experimental groups. Values of $P<0{\cdot}05$ were considered significant.

### RESULTS

### *Response to treatment and pretreatment value of NK activity and ADCC*

No complete or partial responses were

TABLE III.—*Pretreatment level of NK activity and ADCC*

| | Target cell | Normal | A group | Significance* | B group | Significance† |
|---|---|---|---|---|---|---|
| NK | K-562 | 43·0±3·1‡ | 41·1±3·9 | N.S. | 27·4±7·1 | $P<0·05$ |
| ADCC | PC-9 | 43·7±2·2 | 49·5±3·1 | N.S. | 30·4±4·5 | $P<0·02$ |
| NK | PC-9 | 8·7±1·5 | 7·2±0·8 | N.S. | 4·2±0·4 | $P<0·01$ |

* Between normal and A group (patients with stabilized disease).

† Between normal and B group (patients with progressive disease).

‡ Cytotoxicity (mean ± s.e.).

FIG. 3.—Changes in NK activity and ADCC after the beginning of chemotherapy in group A patients (responders). Y.K. was administered 8 mg of CQ by bronchial-artery infusion at 5 weeks.

observed. Eight patients (Group A) experienced stabilized disease and 7 patients (Group B) experienced progressive disease. Three patients with pleural effusions were well controlled by tube drainage and local instillation of anticancer agents. NK activity against K-562, and ADCC and NK activity against PC-9 were not decreased in Group A before treatment. In contrast, in Group B these values were significantly lower than normal, even before treatment (Table III).

*Changes in NK activity and ADCC after the initiation of chemotherapy*

Fig. 3 shows the changes in NK activity and ADCC after the initiation of chemotherapy in 8 patients in Group A. NK activity against K-562, and NK activity and ADCC against PC-9 cells were not decreased 1 week after the initiation of treatment. NK activity against K-562 and PC-9 tended to decrease 2 weeks after the beginning of treatment, and reached the lowest level at 3–4 weeks. However, the activity was partially restored by 5 weeks, and returned to pretreatment levels in 6 weeks, except for Patient Y.K., who received 8 mg of CQ by bronchial-artery infusion. The changes in ADCC after the initiation of chemotherapy were quite similar to those of NK activity. All the patients were dischargeable at the end of the chemotherapy.

Fig. 4 shows the changes in NK activity and ADCC in 7 patients in Group B. All the activities began to decrease 2 weeks after the initiation of treatment. NK activity against PC-9 reached its lowest level at this stage. NK activity against K-562 and ADCC against PC-9 reached their lowest level at 3 and 5 weeks, respectively. None of the activities in this group of patients returned to normal levels, even after 6 weeks, and none of the patients in Group B could be discharged from hospital. All died within 3 months of the initiation of treatment.

FIG. 4.—Changes in NK activity and ADCC after the beginning of chemotherapy in group B patients (progressive disease).

### *Detailed analysis of NK activity and ADCC in 6 patients*

Six patients who administered pepleomycin (PLM) anticancer antibiotic, and CQ were analysed for the effect of the anticancer agents on NK activity and ADCC. They were divided into 3 pairs according to the route and timing of PLM administration. Pair 1 received small divided doses of PLM. Pairs 2 and 3 received large intermittent doses of PLM simultaneously and alternately with CQ, respectively.

Fig. 5 shows the changes in NK activity and ADCC in the patients receiving small divided doses of PLM, and CQ. Patient Y.K. received bronchial-artery infusion of 8 mg of CQ 5 weeks after the initiation of treatment. Response of these patients to treatment was SD. In both cases, small divided doses of PLM alone had no effect on NK activity or ADCC. After the first dose of 6 mg CQ, NK activity against K-562 and PC-9 began to decrease; in contrast, ADCC against PC-9 did not change. One week after the second dose of CQ, ADCC against PC-9 was significantly decreased and NK activity against K-562 and PC-9 reached the lowest level in both cases;

FIG. 5.—Effect of chemotherapy on NK activity and ADCC in patients treated with small divided dose of pepleomycin (PLM) whose disease was stabilized. ●- - -●, NK (K-562); ○—○ ADCC (PC-9); ●—● NK (PC-9). PS, performance status; BAI: bronchial artery infusion.

FIG. 6.—Effect of chemotherapy on NK activity and ADCC in patients treated with large intermittent dose of pepleomycin simultaneous with CQ. ●- - -●, NK (K-562); ○—○, ADCC (PC-9); ●—● NK (PC-9). PS, performance status.

these activities tended to be restored 5 weeks after the initiation of treatment. In Patient M.S. they returned to pretreatment levels in 6 weeks. On the other hand, these activities decreased again 1 week after the bronchial-artery infusion of 8 mg CQ in Patient Y.K. These results suggest that small divided doses of PLM did not reduce NK activity and ADCC, and that the two administrations of 6 mg CQ weekly induced the marked decrease in these activities; in addition, administration of CQ by the intra-arterial route had the same effect as by the i.v. route.

Fig. 6 shows the changes in NK activity and ADCC in patients receiving large intermittent doses of PLM simultaneously with CQ. In Patient C.N., whose response

FIG. 7.—Effect of chemotherapy on NK activity and ADCC in patients treated with large intermittent dose of pepleomycin (alternating with CQ). ●- - -●, NK (K-562): ○—○, ADCC (PC-9): ●—● NK (PC-9).

was SD, PLM and CQ were started simultaneously, and 20 mg of PLM was added in Week 4. On the other hand, 20 mg of PLM preceded the simultaneous combination of PLM and CQ given 3 times in Patient S.S., whose response was PD. As shown in Fig. 6, NK activity and ADCC decreased 1 week after the initiation of treatment, stayed low through the 2nd week, and showed slight recovery by the 3rd week in patient CN. In Patient S.S., 20 mg of i.v. PLM alone had no effect upon NK activity or ADCC. After the beginning of simultaneous administration of PLM and CQ, the activities decreased, and did not return to pretreatment levels.

Fig. 7 shows the changes in NK activity and ADCC in patients receiving large intermittent doses of PLM alternately with CQ. The responses to treatment were SD in H.A., and PD in T.K. The changes in NK activity and ADCC were essentially the same as in Fig. 6. Again PLM alone did not affect either activities, and the administration of CQ apparently decreased both.

### DISCUSSION

In clinical practice, a number of tests are conducted to determine the immune reactivity of the patient (Hersh, 1981; Sone *et al.*, 1977). The principal cell in immunological surveillance against cancer has been thought to be the T cell. However, multiple immunological methods to evaluate the T-cell functions have failed to confirm a major role for the T cell in immunological surveillance, since it is difficult to demonstrate a good correlation between the reactivity of the T cell and the prognosis of the cancer. The role of other potential antitumour effector cells such as NK cells, K cells and macrophages needs more intensive investigation (McCredie *et al.*, 1979; Saijo *et al.*, 1980, 1982*a*). NK cells have been shown to have cytotoxic activity against syngeneic, allogeneic, and xenogeneic target cells without prior sensitization. The characteristics (including surface receptors) of human NK cells have been investigated intensively. On the other hand ADCC is mediated by various leucocyte populations, depending on the target cell. When nucleated target cells are used, ADCC is mediated exclusively by a subpopulation of lymphocytes known as killer or K cells. The K cells have Fc receptors which interact with specific immunoglobulins on the surface of the target cell.

The *in vivo* significance of NK activity and ADCC is still unclear, but these activities seems consistent with the existence of a surveillance mechanism against tumour growth (Menon & Stefani, 1978). The potential role of NK activity and ADCC in human cancer is now under extensive investigation, in spite of the inconsistent levels of NK activity and ADCC in tumour-bearing patients (Eremin *et al.*, 1978; Forbes *et al.*, 1981*a*, *b*; McCredie *et al.*, 1979; Moore & Potter, 1980; Pross & Baines, 1976). In general, anticancer agents are thought to suppress NK activity and ADCC (Saijo *et al.*, 1982*b*). However, the kinetics of the action of anticancer agents against NK activity and ADCC are complicated. Mantovani *et al.* (1978) reported that azathiopurine and cyclophosphamide induced an apparent decrease in NK activity of mouse spleen lymphocytes 2 days after administration of the drug. On the other hand, Santoni *et al.* (1980) demonstrated an elevated NK activity in peritoneal lymphocytes and a decrease in spleen lymphocytes after the administration of Adriamycin. There are not enough data about the effect of anticancer agents on ADCC. McCredie *et al.* (1979) reported that in patients with normal K-cell activity surgery had no effect on ADCC, but in those with low values before surgery there was a maximal decrease at 5 days, with recovery by 15 days, and that radiotherapy caused a marked decrease in ADCC, maximal at 4 weeks, with recovery by about 12 weeks in those who had no known local or distant disease after the completion of treatment.

In this study, it was demonstrated that

NK activity and ADCC in 7 patients with poor prognosis were significantly decreased even before treatment, and that they began to decrease 2 weeks after the initiation of treatment and reached the lowest level at 3–4 weeks in all patients. Thereafter, the activities returned to the pretreatment level in 8 patients with stabilized disease; in contrast, they were not restored in 7 patients with progressive disease and poor prognosis. These results suggest that NK activity and ADCC are valuable prognostic factors in patients with Stage $IIIM_0$ and $IIIM_1$ carcinoma of the lung. In addition, we clearly demonstrated that the effect of chemotherapy on NK activity and ADCC differed according to the drug used. Administration of CQ apparently decreased in NK activity and ADCC, irrespective of the route of administration. On the other hand, the effect of PLM was not clearly detected irrespective of timing, dose or route of administration. It should be stressed that the detailed analysis of the effect of each anticancer agent on NK activity and ADCC is necessary for the establishment of better treatment regimens for advanced carcinoma of the lung (Saal *et al.*, 1977). In spite of the promising results of an attempt to monitor the immune system in patients with Stage $IIIM_0$ and $IIIM_1$ carcinoma of the lung, the phenomena observed in this pilot study still await statistical confirmation in a larger, controlled, more systematic study. The complexities inherent in the clinical situation and chemotherapeutic regimens make it premature to engage in detailed speculation about the precise role played by the circulating cytolytic lymphocytes. In addition, plastic non-adherent cells were used as effector cells of NK and ADCC assay throughout this study. The composition of the lymphocyte population in the blood is an essential factor for further assessment of the significance of these data. Nevertheless, we are encouraged by having a test available which may give reproducible information on chemotherapy induced effects on immune reactivity and which may correspond to the course of the neoplastic disease.

This work was supported by Grants-in-Aid for Cancer Research from the Ministry of Health and Welfare and from the Ministry of Education, as well as by a grant from the Science and Technology Agency.

The authors gratefully acknowledge the kind advice and support of Professor Hisanobu Niitani of Nippon Medical School, and Drs Akira Ozaki and Yasuo Beppu.

### REFERENCES

BOYUM, A. (1968) Separation of leukocytes from blood and bone marrow. *Scand. J. Clin. Lab. Invest.* **21** (Suppl. 97), 77.

EREMIN, O., ASHBY, J. & STEPHENS, J. P. (1978) Human natural cytotoxicity in the blood and lymphoid organs of healthy donors and patients with malignant disease. *Int. J. Cancer*, **21**, 35.

FORBES, J. T., GRECO, F. A. & OLDHAM, R. K. (1981*a*) Human natural cell-mediated cytotoxicity. II. Levels in neoplastic disease. *Cancer Immunol. Immunother.*, **11**, 147.

FORBES, J. T., NIBLACK, G. D., FUCHS, R., RICHIE, R. E., JOHNSON, H. K. & OLDHAM, R. K. (1981*b*) Human natural cell-mediated cytotoxicity. I. Levels in peripheral blood, cord blood, and thoracic duct lymphocytes. *Cancer Immunol. Immunother.*, **11**, 139.

HABU, S., FUKIMU, H., SHIMAMURA, K. & 4 others (1981) *In vivo* effects of anti-asialo GMI. I. Reduction of NK activity and enhancement of transplanted tumor growth in nude mice. *J. Immunol.*, **127**, 34.

HERBERMAN, R. B. & HOLDEN, H. T. (1979) Natural killer cells as antitumour effector cells. *J. Natl Cancer Inst.*, **62**, 441.

HERSH, E. M., MURPHY, S. G., QUESADA, J. R., GUTTERMAN, J. U., GASCHWIND, C. R. & MORGAN J. (1981) Effect of immunotherapy with *Corynebacterium parvum* and methanol extraction residue of BCG administered intravenously on host defense function in cancer patients. *J. Natl Cancer Inst.*, **66**, 993.

MANTOVANI, A., LUINI, W., PERI, G., VECCHI, A. & SPREAFICO, F. (1978) Effect of chemotherapeutic agents on natural cell-mediated cytotoxicity in mice. *J. Natl Cancer Inst.*, **61**, 1255.

MCCREDIE, J. A., MACDONALD, H. R. & WOOD, S. B. (1979) Effect of operation and radiotherapy on antibody-dependent cellular cytotoxicity. *Cancer*, **44**, 99.

MENON, M. & STEFANI, S. S. (1978) Lymphocyte mediated natural cytotoxicity in neoplasia. *Oncology*, **35**, 63.

MOORE, M. & POTTER, M. R. (1980) Enhancement of human natural cell-mediated cytotoxicity by interferon. *Br. J. Cancer*, **41**, 378.

PROSS, H. F. & BAINES, M. Q. (1976) Spontaneous human lymphocyte-mediated cytotoxicity against tumor target cells. I. The effect of malignant disease. *Int. J. Cancer*, **18**, 593.

SAAL, J. G., RIETHMÜLLER, G., RIEBER, E. P., HADAN, M., EHINGER, H. & SCHNEIDER, W. (1977)

Regional BCG-therapy of malignant melanoma: *In vitro* monitoring of spontaneous cytolytic activity of circulating lymphocytes., *Cancer Immunol. Immunother.*, **3**, 27.

SAIJO, N., IRIMAJIRI, N., OZAKI, A., SHIMIZU, E. & NIITANI, H. (1980) Effectors of BCG and ACNU on the cytostatic activity of macrophages in normal and tumour-bearing rats., *Br. J. Cancer*, **42**, 162.

SAIJO, N., KIMURA, K., IRIMAJIRI, N., OZAKI, A., HASHIMOTO, N. & NIITANI, H. (1981) Antitumor immune response in tumor-bearing patients: Natural killer and antibody-dependent cellular cytotoxicity. *Gan To Kagakuryoho*, **8**, 2.

SAIJO, N., SHIMIZU, E., IRIMAJIRI, N. & 4 others (1982*a*) Analysis of natural killer activity and antibody-dependent cellular cytotoxicity in healthy volunteers and in patients with primary lung cancer and metastatic pulmonary tumors. *J. Cancer Res. Clin. Oncol.* **102**, 195.

SAIJO, N., IRIMAJIRI, N., OZAKI, A. & 4 others (1982*b*) Effect of BCG and cyclophosphamide on the spontaneous and antibody-dependent cytotoxicity of peritoneal and spleen lymphocytes of ACI/N rats. *Gann*, **73**, 270.

SANTONI, A., RICCARDI, C. & HERBERMAN, R. G. (1980) Effect of Adriamycin on the activity of mouse Natural Killer cells. *J. Immunol.*, **124**, 2329.

SONE, S., YATA, K. & TSUBURA, E. (1977) Rebound phenomenon of phytohemogglutinin skin reactivity in leukemia and malignant lymphoma after chemotherapy. *Gann*. **68**, 483.